# Whole-transcriptome analysis and construction of an anther development-related ceRNA network in Chinese cabbage (*Brassica campestris* L. ssp. *pekinensis*)

**DOI:** 10.1038/s41598-022-06556-2

**Published:** 2022-02-17

**Authors:** Fengyan Shi, Zhijin Pang, Chuanhong Liu, Li Zhou, Chong Tan, Jie Ren, Xueling Ye, Hui Feng, Zhiyong Liu

**Affiliations:** 1 Key Laboratory of Protected Horticulture (Shenyang Agricultural University), Ministry of Education, 120 Dongling Road, Shenhe District, Shenyang, 110866 China; 2 Integrated Technical Service Center, Bayuquan Customs, Yingkou, 115007 China

**Keywords:** Molecular biology, Plant sciences

## Abstract

Anther development is precisely regulated by a complex gene network, which is of great significance to plant breeding. However, the molecular mechanism of anther development in Chinese cabbage is unclear. Here, we identified microRNAs (miRNAs), mRNAs, long non-coding RNAs (lncRNAs), and circular RNAs (circRNAs) related to anther development in Chinese cabbage (*Brassica campestris* L. ssp. *pekinensis*) to construct competitive endogenous RNA (ceRNA) regulatory networks and provide valuable knowledge on anther development. Using whole-transcriptome sequencing, 9055, 585, 1344, and 165 differentially expressed mRNAs (DEmRNAs), miRNAs (DEmiRNAs), lncRNAs (DElncRNAs), and circRNAs (DEcircRNAs) were identified, respectively, in the anthers of Chinese cabbage compared with those in samples of the vegetative mass of four true leaves. An anther-related ceRNA regulatory network was constructed using miRNA targeting relationships, and 450 pairs of ceRNA relationships, including 97 DEmiRNA–DEmRNA, 281 DEmiRNA–DElncRNA, and 23 DEmiRNA–DEcircRNA interactions, were obtained. We identified important genes and their interactions with lncRNAs, circRNAs, and miRNAs involved in microsporogenesis, tapetum and callose layer development, pollen wall formation, and anther dehiscence. We analyzed the promoter activity of six predominant anther expression genes, which were expressed specifically in the anthers of *Arabidopsis thaliana*, indicating that they may play an important role in anther development of Chinese cabbage. This study lays the foundation for further research on the molecular mechanisms of anther growth and development in Chinese cabbage.

## Introduction

Anther development is an important biological process for male fertility and sexual reproduction in plants; this complex process includes several genes that participate in signal transduction, cell division, apoptosis, metabolism, and transportation, which are activated or inhibited by spatially and temporally fine-tuned regulatory pathways^1,2^. Chinese cabbage (*Brassica campestris* L. ssp. *pekinensis*) is a typical allogamous *Brassica* crop, which displays significant heterosis. The study of anther development is important for Chinese cabbage breeding. Though several genes related to anther development have been identified in Chinese cabbage, such as *BAN103*^3^, *BcMS2*^4^, *BcMF2*^5^, *BcMF5*^6^, *BcMF6*^7^, *BrSKS13*^8^, *BcMF12*^9^, *BcMF13*^10^, *BcMF19*^11^, and *BcMF20*^12^, the molecular mechanism of anther development remains unstudied.

In recent years, with the rapid development of high-throughput sequencing technology, genome-wide differential RNA expression analysis has allowed whole-transcriptome level studies on anther development in flowering plants^13,14^. Messenger RNA (mRNA) is a single-stranded RNA transcribed from a strand of DNA as a template, which carries genetic information to guide protein synthesis. In addition to mRNA, cells also contain various types of non-coding RNAs (ncRNAs), such as small RNAs with regulatory functions [represented by microRNA (miRNA), long non-coding RNAs (lncRNAs), and circular RNAs (circRNAs)]; the regulatory objects of these ncRNAs are related to the mRNA expression.

miRNAs are endogenous ncRNAs (18–25 nucleotides) with regulatory functions and found in eukaryotes^15^. Recent studies have demonstrated that miRNAs play an important regulatory role in the development of flower organs. For example, two novel miRNAs (novel-miR-448 and novel-miR-335) that are specifically expressed in flower buds were identified in Chinese cabbage, and their targeted sucrose transporters, SUC1 and H(+)-ATPase 6, were found to be inhibited, possibly leading to an energy deficiency and pollen abortion^16^. lncRNA is a type of ncRNA, exceeding 200 nt in length, and mainly transcribed from the antisense chain and spacer region of the protein-coding gene. lncRNAs are involved in many important regulatory processes such as genomic imprinting, chromatin modification, transcriptional activation, transcriptional interference, and nuclear transport^17,18^. It has been reported that several lncRNAs activate their neighboring genes through cis-mediated mechanisms. Several lncRNAs also act as sponges to compete with endogenous miRNAs, thus regulating the target genes of miRNAs^19,20^. In recent years, lncRNAs related to anther development have been identified in several plants such as rice, trilobite oranges, and diploid strawberries^18,21,22^. Covalently closed, single-stranded circRNA is a special endogenous ncRNA, which has no free 5' cap structure or 3' poly (A) structure and is insensitive to nuclease; therefore, it is more stable than ordinary linear RNA. Owing to the development of high-throughput sequencing techniques and bioinformatics, thousands of circRNAs have been identified in cells and tissues of different species, most of which are conserved and stable among different species. The expression of circRNAs is cell-, tissue-, and developmental stage-specific. Although many circRNAs have been identified, research on the function(s) of circRNAs and the mechanism of circRNA formation is in its early stages^23,24^. Several studies have demonstrated that circRNAs can be used as competitive endogenous RNAs (ceRNAs) to communicate with and regulate each other at the post-transcriptional level through competitive shared miRNA response elements^25,26^.

Recent studies have demonstrated that the interactions among lncRNAs, circRNAs, miRNAs, and pseudogene transcripts can affect gene expression, and accordingly, a ceRNA hypothesis has been proposed^27^. Increasing evidence has demonstrated that the ceRNA network plays an important regulatory role in many plants. For instance, a ceRNA network was found in *Populus*, revealing its role in adaptation to low nutrition^28^. Fan et al.^29^ constructed a ceRNA network to elucidate the potential mechanism of Paulownia witches’ broom disease by analyzing the expression of miRNAs, lncRNAs, circRNAs, and mRNAs. In rice, a ceRNA network was constructed, which may play an important role in regulating the response of phosphate-starved plants^30^. However, the regulatory network of ceRNA in the anther development of Chinese cabbage has not yet been established, and the interactions among circRNAs, lncRNAs, miRNAs, and mRNAs in anther development require further study.

In this study, we performed whole-transcriptome sequencing of anthers samples (‘Ant’) and samples of the vegetative mass of four true leaves (‘Mix’), identified changes in the expression of miRNAs, lncRNAs, circRNAs, and mRNAs, and analyzed their potential regulatory roles using the Gene Ontology (GO) and Kyoto Encyclopedia of Genes and Genome (KEGG) databases. Additionally, a ceRNA network was established, which lays the foundation for exploring the potential regulatory mechanism of Chinese cabbage anther development.

## Results

### Identification of miRNAs and their functions

To fully understand the miRNA repertoire related to anther development in Chinese cabbage, ‘Ant’ and ‘Mix’ sRNA libraries were constructed and sequenced. A total of 24,706,757 and 22,291,255 raw reads were obtained from the ‘Ant’ and ‘Mix’ sRNA libraries, and after filtering, 13,351,418 and 12,257,469 valid reads were obtained, respectively (Table S1). Based on original sequencing data analysis and statistics, we calculated the length distribution of the filtered valid data to be distributed in the range of 20–24 nt, which conforms to the typical characteristics of Dicer enzyme cutting (Table S2). A total of 1360 miRNAs (709 known and 651 novel) were identified (Table S3). Family analysis of detected miRNAs to explore the existence of miRNA families in other species aids in understanding the conservation of miRNAs in evolutionary relationships. A total of 65 miRNA families conserved in plants were identified in this study (Table S4).

**Figure 1:**
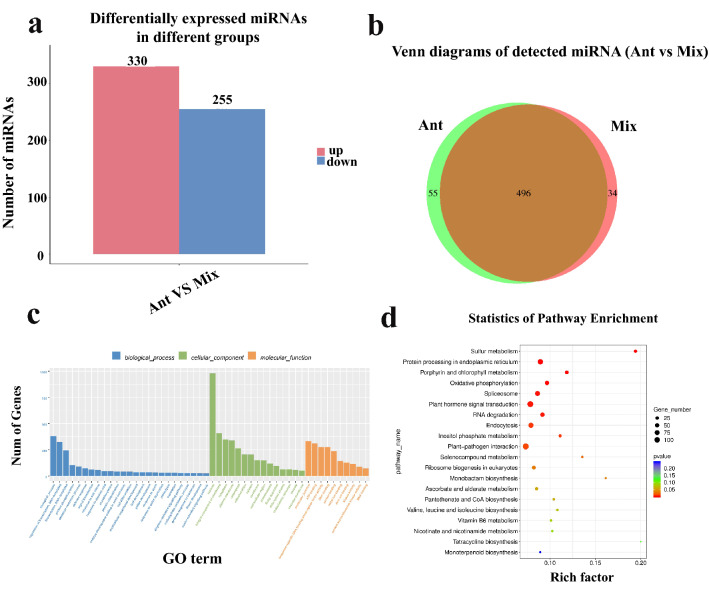
Identification and analysis of differentially expressed miRNAs (DEmiRNAs) between anther samples (‘Ant’) and samples of the vegetative mass of four true leaves (‘Mix’). (**a**) Statistical analysis of the number of upregulated and downregulated DEmiRNAs identified between ‘Ant’ and ‘Mix’; (**b**) Venn diagram displaying the number of DEmiRNAs in ‘Ant’ and ‘Mix’; (**c**) Gene Ontology (GO) classifications of DEmiRNAs; (**d**) Kyoto Encyclopedia of Genes and Genome (KEGG) pathway assignments of DEmiRNAs.

To identify miRNAs, that responded to anther development, and their expression patterns, we compared the ‘Ant’ and ‘Mix’ libraries. A total of 585 DEmiRNAs were obtained, including 330 (166 known and 164 novel) upregulated and 255 (198 known and 57 novel) downregulated miRNAs (Table S5, Fig. 1a). Additionally, 55 and 34 DEmiRNAs with specific expression were identified in ‘Ant’ and ‘Mix’ libraries, respectively (Fig. 1b). We predicted 3687 target genes for the 585 DEmiRNAs (Table S6).

To further understand the potential function of miRNAs, the target genes of miRNAs were analyzed using GO and KEGG (Tables S7 and S8). GO annotation revealed that most DEmRNAs were annotated to the ‘biological process’ (BP) ontology. Additionally, common annotations were ‘regulation of transcription, DNA-templated,’ ‘transcription’, and ‘protein phosphorylation’ in the BP ontology; ‘nucleus,’ ‘integral component of membrane’, ‘cytoplasm’, and ‘plasma membrane’ in the ‘cellular component’ (CC) ontology; and ‘molecular function’, ‘ATP binding’, ‘sequence-specific DNA binding transcription factor activity’, and ‘DNA binding’ in the ‘molecular function’ (MF) ontology (Fig. 1c). KEGG enrichment analysis revealed that the targets of DEmiRNAs were significantly enriched in several pathways that may be related to anther development, including ‘plant hormone signal transduction’, which plays an important regulatory role in plant anther development^31^, ‘endocytosis’, which is essential for the development of male reproductive organs in *Arabidopsis*^32^, ‘plant–pathogen interaction’, which plays a dominate role in recessive genic male sterility of cabbage^33^, ‘protein processing in the endoplasmic reticulum’, and ‘oxidative phosphorylation’, which were also significantly enriched in the comparative transcriptome analysis of tobacco *sua*-cytoplasmic male sterility, indicating that they may be involved in anther development^34^ (Fig. 1d).

### Identification of lncRNAs and mRNAs and their functions

We performed whole-transcriptome sequencing of the ‘Ant’ and ‘Mix’ RNA libraries using the Illumina Hiseq 2500 platform. High-quality clean reads were used to identify mRNAs, lncRNAs, and circRNAs differentially expressed in response to anther development. A total of 139,573,514 and 141,748,652 raw reads were obtained; filtering resulted in 136,757,604 (97.98%) and 139,969,178 (98.74%) valid reads from the ‘Ant’ and ‘Mix’ RNA libraries, respectively. After removing the rRNA genes, the valid reads were mapped to the *Brassica rapa* v3.0 reference genome. The percentage of mapped valid reads in the ‘Ant’ and ‘Mix’ libraries were 89.00% and 92.06%, respectively (Table S9).

**Figure 2:**
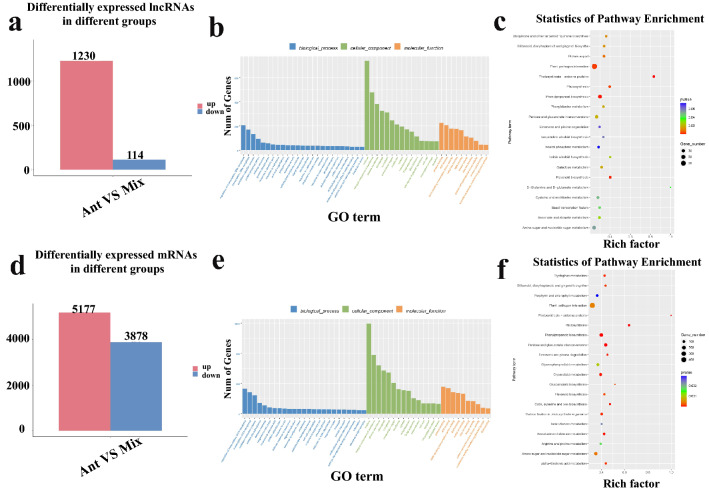
Identification and analysis of differentially expressed lncRNAs (DElncRNAs) and mRNAs (DEmRNAs) between anther samples (‘Ant’) and samples of the vegetative mass of four true leaves (‘Mix’). **(a**) Statistics of the number of upregulated and downregulated DElncRNAs identified between ‘Ant’ and ‘Mix’; (**b**) Gene Ontology (GO) classifications of DElncRNAs; (**c**) Kyoto Encyclopedia of Genes and Genome (KEGG) pathway assignments of DElncRNAs; (**d**) Statistical analysis of the number of upregulated and downregulated DEmRNAs identified between ‘Ant’ and ‘Mix’; (**e**) GO classifications of DEmRNAs; (**f**) KEGG pathway assignments of DEmRNAs.

The mRNA and lncRNA expression levels were estimated using the FPKM value of Cuffdiff. A total of 2384 lncRNAs and 33,821 mRNAs were identified (Table S3). Compared with those of ‘Mix,’ 1344 lncRNAs and 9055 mRNAs were differentially expressed in the anthers of Chinese cabbage. Among the DElncRNAs and DEmRNAs, 1230 lncRNAs and 5177 mRNAs were upregulated, while 114 lncRNAs and 3878 mRNAs were downregulated in the anther (Table S5, Fig. 2a, d). Next, we compared the identified lncRNAs with the genomic characteristics of Chinese cabbage protein-coding genes. The structural characteristics and expression levels of lncRNAs and mRNAs were significantly different (Fig. S1). Most transcript lengths of mRNA were longer than 1000 bp, while those of lncRNAs were less than 500 bp. The average ORF length of mRNAs was longer than that of lncRNAs. The ORF length of most lncRNAs was 0–50 aa, while the ORF length of most mRNAs was 100–500 aa. Compared with those of mRNAs, lncRNAs had fewer exons, on average, and most lncRNAs had only one or two exons. Additionally, mRNA expression levels were higher than those of lncRNAs.

GO enrichment and KEGG analyses were performed to explore the functions of the DEmRNAs (Tables S7 and S8). DEmRNAs were commonly annotated to the GO terms ‘regulation of transcription, DNA-templated’, ‘oxidation–reduction process’, ‘protein phosphorylation’, and ‘defense response’ in the BP ontology; ‘nucleus’, ‘integral component of membrane’, ‘plasma membrane’, and ‘chloroplast’ in the CC ontology; and ‘protein binding’, ‘ATP binding’, ‘DNA-binding transcription factor activity’, and ‘DNA binding’ in the MF ontology (Fig. 2e). Additionally, many GO terms related to pollen development, including ‘pollen tube growth’, ‘carbohydrate-binding’, ‘plant-type cell wall’, ‘lipid transport’, and ‘cell wall modification’, were significantly enriched. The KEGG analysis revealed that the following pathways were highly enriched in the DEmRNAs: ‘photosynthesis’, ‘photosynthesis-antenna proteins’, ‘phenylpropanoid biosynthesis’, ‘glucosinolate biosynthesis’, ‘pentose and glucuronate interconversions’, ‘glycerophospholipid metabolism’, ‘amino sugar and nucleotide sugar metabolism’, ‘cutin, suberin, and wax biosynthesis’, ‘glycerolipid metabolism’, and ‘alpha-Linolenic acid metabolism’ (Fig. 2f). These GO terms and KEGG pathways of DEmRNAs may be involved in anther development in Chinese cabbage.

**Figure 3:**
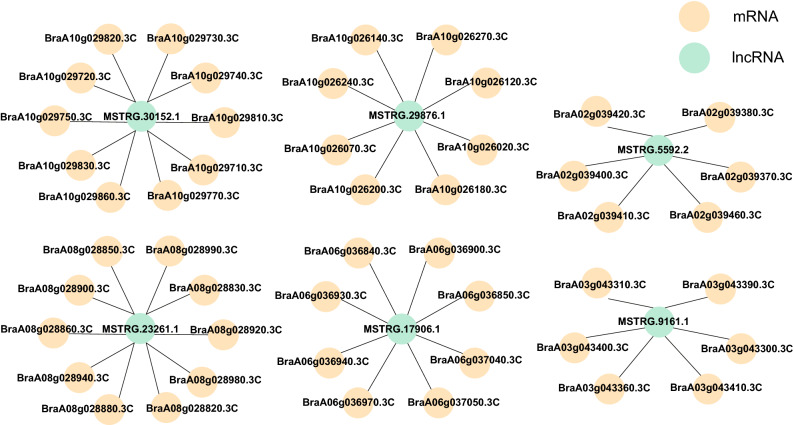
Multiple differentially expressed mRNAs interacted with one differentially expressed lncRNA. Lines indicate interactions.

lncRNAs can cis-regulate neighboring genes to regulate the expression of transcriptional or post-transcriptional genes^35,36^. lncRNA cis-regulated target genes were predicted based on positional relationships. We searched for lncRNAs and mRNAs with differential expressions 100 Kb upstream and downstream of the chromosomes; these lncRNAs were considered cis-regulated. By analyzing the cis-regulatory function of lncRNAs, a co-expression network of DElncRNAs and DEmRNAs was constructed. Most DEmRNAs and DElncRNAs were one-to-many matching, while several were one-to-one matching (Table S10). Considering that the graph cannot display much network regulation information between lncRNAs and mRNAs, more mRNAs for co-expression with lncRNAs were selected to establish their interaction network graph. As displayed in Fig. 3, both *MSTRG.30152.1* and *MSTRG.23261.1* interacted with ten mRNAs; *MSTRG.29876.1* and *MSTRG.17906.2* interacted with eight mRNAs; and *MSTRG.9161.1* and *MSTRG.5592.2* interacted with six mRNAs.

GO and KEGG enrichment analyses of genes targeted by DElncRNAs were performed (Tables S7 and S8). Similar to DEmRNAs, common GO terms of DElncRNAs were ‘nucleus,’ ‘integral component of membrane,’ ‘plasma membrane’, ‘cytoplasm’, ‘chloroplast’, ‘protein binding’, ‘regulation of transcription’, ‘DNA-templated’, and ‘ATP binding’ (Fig. 2b). We analyzed the KEGG enrichment of the target genes of DElncRNAs and found 127 pathways, of which 16 were significantly enriched KEGG metabolic pathways, including ‘photosynthesis-antenna proteins’, ‘phenylpropanoid biosynthesis’, ‘flavonoid biosynthesis’, ‘plant–pathogen interaction’, ‘photosynthesis’, ‘protein export’, ‘pentose and glucuronate interconversions’, and ‘amino sugar and nucleotide sugar metabolism’, which may be involved in the regulation of anther development (Fig. 2c).

### Identification of circRNAs and their functions

**Figure 4:**
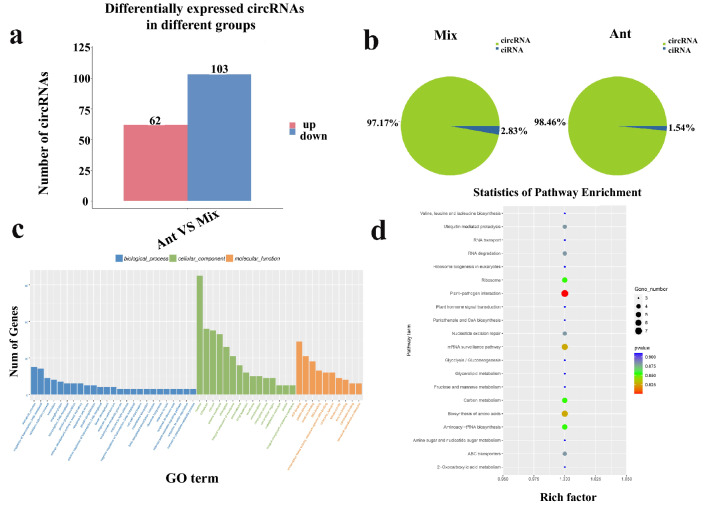
Identification and analysis of differentially expressed circRNAs (DEcircRNAs) between anther samples (‘Ant’) and samples of the vegetative mass of four true leaves (‘Mix’). (**a**) Statistical analysis of the number of upregulated and downregulated DEcircRNAs identified between ‘Ant’ and ‘Mix’; (**b**) circRNAs of ‘Ant’ and ‘Mix’; (**c**) Gene Ontology classifications of DEcircRNAs; (**d**) Kyoto Encyclopedia of Genes and Genome (KEGG) pathway assignments of DEcircRNAs.

A total of 106 and 65 circRNAs were identified from the ‘Ant’ and ‘Mix’ RNA libraries, respectively (Table S3). We identified 165 DEcircRNAs, including 62 upregulated and 103 downregulated circRNAs (Table S5, Fig. 4a). circRNAs composed of exons or introns were counted using the CIRCexplorer2 software. The results revealed that more than 97% of the circRNAs were full-exon circRNAs (Fig. 4b).

To date, a large number of circRNAs have not been functionally annotated^37^. To further understand the functions of circRNAs in Chinese cabbage, we conducted GO and KEGG analyses of the differentially expressed circRNA-hosting genes (Tables S7 and S8). The circRNA-hosting genes were mainly enriched in the GO terms ‘nucleus process’, ‘cytoplasm’, ‘cytosol’, ‘plasma membrane’, ‘ATP binding’, ‘chloroplast’, ‘protein binding’, ‘integral component of membrane’, ‘metal ion binding’, and ‘plasmodesma’ (Fig. 4c). The KEGG enrichment analysis revealed that most circRNA-hosting genes were enriched in the terms ‘plant-pathogen interactions’, ‘mRNA surveillance pathways’, ‘biosynthesis of amino acids’, ‘aminoacyl-tRNA biosynthesis’, ‘carbon metabolism’, ‘ribosome’, ‘nucleotide excision repair’, ‘ubiquitin-mediated proteolysis’, ‘RNA degradation’, and ‘ABC transporter pathways’, which laid a foundation for exploring the function of circRNAs in Chinese cabbage anthers (Fig. 4d).

### Constructing a ceRNA–miRNA–target gene regulatory network related to anther development in Chinese cabbage

**Figure 5:**
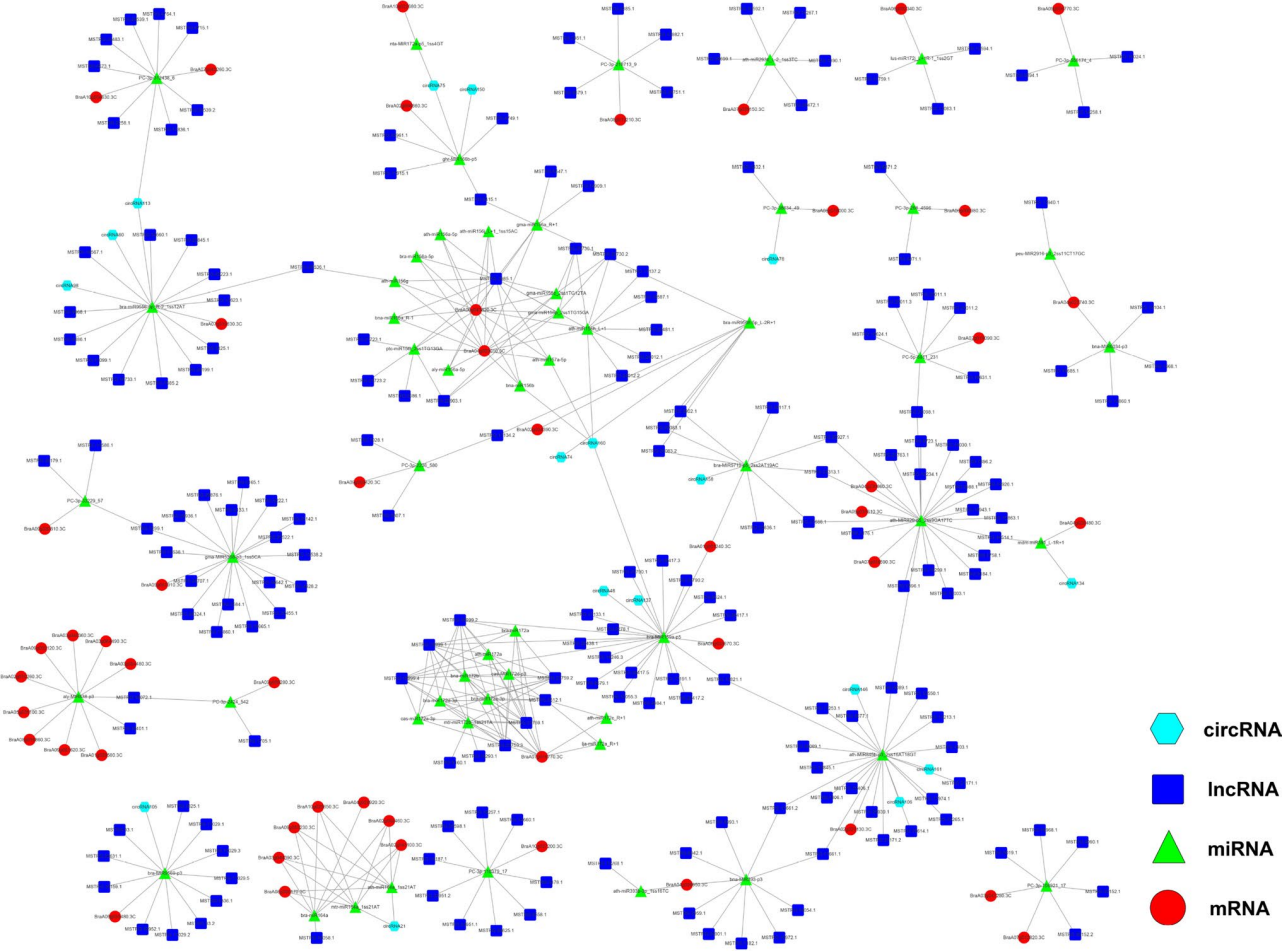
CeRNA network constructed using anther development-related differentially expressed mRNAs, lncRNAs, circRNAs, and miRNAs.

It has been reported that lncRNAs and circRNAs can interact with miRNAs through miRNA reaction elements in the ceRNA network^27^. To reveal the global regulatory network of protein-coding RNAs and ncRNAs that are related to anther development based on gene annotation, the DEmRNAs that play an important role in anther development were selected to establish the ceRNA–miRNA–target gene regulatory network based on the ceRNA theory consisting of 49 DEmRNAs, 55 DEmiRNAs, 196 DElncRNAs, and 17 DEcircRNAs. We established candidate ceRNA relationships through targeting miRNA relationships and obtained 450 pairs of ceRNA relationships, including 97 DEmiRNA–DEmRNA, 281 DEmiRNA–DElncRNA, and 23 DEmiRNA–DEcircRNA interactions (Table S11). Perl script was used to integrate lncRNA–miRNA–mRNA and circRNA–miRNA–mRNA networks, and the Cytoscape software (https://cytoscape.org) was used to visualize the regulatory relationship (Fig. 5). In the ceRNA network, the target genes were mainly involved in plant hormone signal transduction, starch and sucrose metabolism, pentose and glucuronate interconversions, RNA degradation, plant–pathogen interactions, carbon metabolism, pyruvate metabolism, and carbon fixation in photosynthetic organisms, based on the KEGG analysis (Table S11). This advanced the understanding of the mechanism of anther development in Chinese cabbage.

Some important transcription factors, enzymes, and regulatory proteins involved in anther and pollen development were identified in this study. These included AP2-like ethylene-responsive transcription factors (*BraA01g001240.3C,* regulated by 27 DElncRNAs, four DEcircRNAs, and two DEmiRNAs; *BraA07g016770.3C,* regulated by nine DElncRNAs and ten DEmiRNAs). It has been reported that the expression of many AP2 genes in anther deficient mutants also decreased, which may be involved in the regulation of anther development^38^. Other transcription factors, enzymes, and regulatiry proteins included ethylene-responsive transcription factors (*BraA09g056770.3C,* regulated by three DElncRNAs and one DEmiRNA; *BraA08g016860.3C,* regulated by two DElncRNAs and one DEmiRNA; *BraA02g036660.3C,* regulated by four DElncRNAs, two DEcircRNAs, and one DEmiRNA), which play an important role in the programmed cell death of papilla cells, compatible pollination, and reproductive growth in *Brassica rapa*^39^; phosphoenolpyruvate carboxylase (PEPC) (*BraA04g029950.3C,* regulated by ten DElncRNAs and two DEmiRNA), which is involved in accelerating the accumulation of storage substances during pollen maturation^40^; squamosa promoter-binding-like protein (SPL) (*BraA04g003010.3C* and *BraA06g043820.3C,* regulated by 15 DElncRNAs, one DEcircRNA, and 13 DEmiRNAs), which is a plant specific transcription factor involved in anther development^41^; and nitrate reductase (*BraA02g024390.3C,* regulated by five DElncRNAs, one DEcircRNA, and one DEmiRNA), whose deficiency has an important effect on flower transformation and nitric oxide release during flower development in *Arabidopsis thaliana*^42^. The above were identified in the ceRNA network, indicating their specific synergistic regulation roles in anther development. More importantly, several well-studied miRNA–target gene pairs related to anther development were identified in this network, including miR156-SPL, miR159-AP2, and miR164-NAC domain-containing protein pairs.

The following important genes and their interactions with lncRNAs, circRNAs, and miRNAs in the main anther development process of Chinese cabbage were also identified in this study: *BraA06g035480.3C*, which is involved in microsporogenesis; *BraA09g009280.3C*, *BraA04g028920.3C*, *BraA10g022680.3C*, *BraA07g040260.3C*, *BraA03g047280.3C*, *BraA06g033670.3C*, *BraA08g018000.3C*, *BraA07g012820.3C*, *BraA04g026480.3*C, and *BraA07g018590.3C*, which affect tapetum development; *BraA06g000980.3C*, *BraA02g023130.3C*, *BraA10g029650.3C*, *BraA03g044390.3C*, *BraA02g043100.3C*, *BraA08g000170.3C*, *BraA05g031610.3C*, and *BraA02g002460.3C*, which are involved in pollen wall development; and *BraA01g001240.3C*, *BraA07g016770.3C*, *BraA04g023740.3C*, *BraA04g030860.3C*, *BraA09g002120.3C*, *BraA09g053230.3C*, *BraA10g004630.3C*, *BraA05g035100.3C*, *BraA09g032810.3C*, *BraA06g002340.3C*, *BraA07g039150.3C*, *BraA02g019090.3C*, and *BraA09g053910.3C*, which affect anther dehiscence. Our results revealed that the regulatory network of ceRNA–miRNA–target genes may be involved in anther development in Chinese cabbage. Further functional studies of ceRNAs, miRNAs, and target genes will help us better understand the mechanisms of anther development and sexual reproduction.

### Verification of RNA-Seq results

To confirm the quality of the RNA-Seq and the expression patterns of miRNAs, lncRNAs, circRNAs, and mRNAs in the ‘Ant’ and ‘Mix’ groups, 13 DEmRNAs (eight known mRNAs and five novel mRNAs), seven DEmiRNAs (five known miRNAs and two novel miRNAs), seven DElncRNAs, and seven DEcircRNAs were randomly selected for qRT-PCR (Fig. S2). Except for *MSTRG.1758.1* and *MSTRG.6184.1*, the expression patterns of other genes were concordant with the RNA-Seq data, suggesting that the RNA-Seq results were reliable. Then, the expression patterns of six predominant anther expression genes (*BraA09g005520.3C*, *BraA01g044980.3C*, *BraA06g036460.3C*, *BraA05g041890.3C*, *BraA04g030880.3C*, and *BraA02g023130.3C*) in different parts of flower organs, including sepals, petals, pistils, anthers, and filaments, were analyzed. The expression levels of these six genes in the anthers were significantly higher than those in the other parts, indicating that these six genes may play an important role in anther development (Fig. S3).

### Promoter activity analysis of predominant anther expression genes

**Figure 6:**
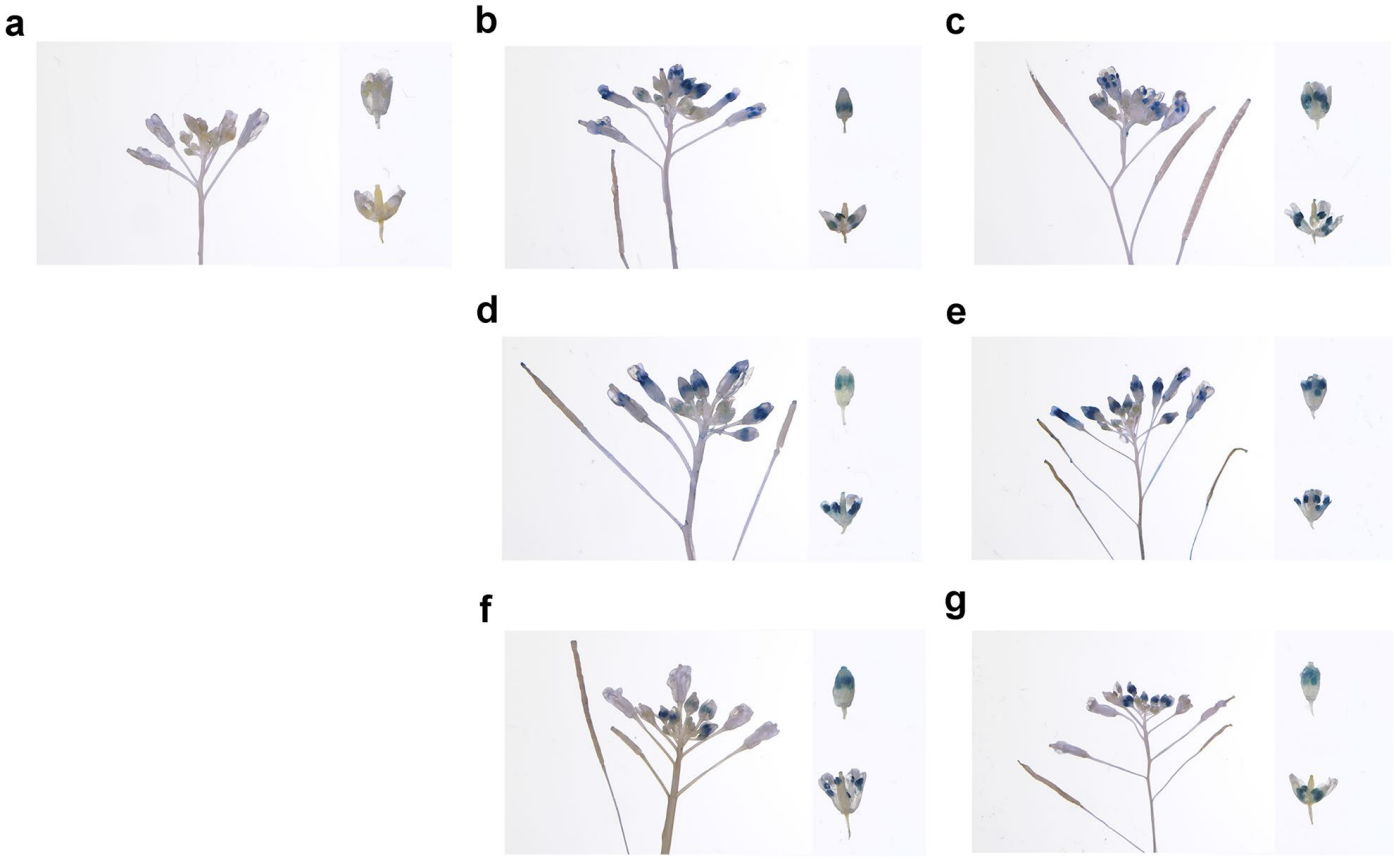
Promoter-GUS activity in wild type and control (**a**), *BraA09g005520.3C* (**b**), *BraA01g044980.3C* (**c**), *BraA06g036460.3C* (**d**), *BraA05g041890.3C* (**e**), *BraA02g023130.3C* (**f**), and *BraA04g030880.3C* (**g**).

To further explore the function of the identified genes that may be involved in Anther development and analyze their promoter activity, we selected six predominant anther expression genes, including four unknown functional genes (*BraA09g005520.3C*, *BraA01g044980.3C*, *BraA06g036460.3C*, and *BraA04g030880.3C*), one encoding polcalcin Bra n 2-like protein (*BraA05g041890.3C*), and one encoding GDSL esterase/lipase (*BraA02g023130.3C*). RNA-Seq data and qRT-PCR revealed that the expression of these genes in ‘Ant’ was much higher than that in ‘Mix’ (Fig. S2), and their expression levels in the anther were significantly higher than those in other floral organs (Fig. S3). Their detailed spatial expression patterns were examined in *Arabidopsis* plants expressing the promoter-driven β-glucuronidase gene. Histochemical staining demonstrated that GUS signals of the six genes only appeared in the anther (Fig. 6), suggesting that *BraA09g005520.3C*, *BraA01g044980.3C*, *BraA06g036460.3C*, *BraA05g041890.3C*, *BraA04g030880.3C*, and *BraA02g023130.3C* are anther specific genes and may perform important functions in regulating anther development.

## Discussion

lncRNAs and circRNAs have been reported to act as ceRNAs and regulate each other by competitive binding to common miRNA response elements. The ceRNA regulatory network formed by lncRNAs, circRNAs, miRNAs, and mRNAs through miRNA response elements is of great significance to post-transcriptional gene regulation in various physiological and pathophysiological processes^43^. In the ceRNA network, miRNAs play an important role in the connection and regulation of different ceRNAs (lncRNAs and circRNAs). The mRNAs can be translated into proteins with direct functions, while lncRNAs and circRNAs can indirectly affect the expression of mRNAs by competitively binding to common miRNAs^27^. Anther development is an important biological process in plant reproduction, and its gene regulatory network has been gradually revealed. However, a comprehensive identification and analysis of lncRNAs and circRNAs as ceRNAs in the anthers of Chinese cabbage is unavailable. To investigate the function of lncRNAs and circRNAs in anther development in Chinese cabbage, we performed sequencing and whole-transcriptome analysis of lncRNAs, circRNAs, miRNAs, and mRNAs from the vegetative mass of four true leaves (‘Mix’) and anther (‘Ant’) samples. A total of 9055 mRNAs, 585 miRNAs, 1344 lncRNAs, and 165 circRNAs were identified as differentially expressed in the anthers.

Compared with mRNAs, lncRNAs have fewer exons, lower expression levels, and shorter transcript lengths. To date, most of the functions of lncRNAs are not fully understood. Co-expression networks of lncRNAs and mRNAs aid in predicting the lncRNA function^44^. By analyzing the cis-regulatory function of lncRNAs, a co-expression network of lncRNAs and mRNAs was constructed. Most mRNAs and lncRNAs were one-to-many matched, but several were one-to-one matched, similar to those in cucumber^45^. It has been demonstrated that circRNAs play an important role in regulating miRNA-mediated post-transcriptional gene expression^46^. In this study, all 165 circRNAs identified were novel circRNAs, including 62 upregulated and 103 downregulated circRNAs in the anther. Here, we first constructed a ceRNA–miRNA–target gene regulatory network related to anther development in Chinese cabbage based on ceRNA theory to obtain 450 pairs of ceRNA relationships. Our findings provide a systematic recognition of mRNAs and ncRNAs and lay the foundation for further work on the regulation mechanism of anther development and the utilization of hybrid breeding in Chinese cabbage.

Anthers are important male reproductive organs of plants that can produce pollen grains. Anther development is a complex process, involving a series of biological events^46^. During the normal development of microspores, many nutrients, including sugars, starch, amino acids, and proteins, play an important role in the development of anthers^47^. In the anther development-related ceRNA network, *BraA06g033670.3C* (F-box protein), *BraA02g023130.3C* (GDSL esterase/lipase), and *BraA04g023740.3C* (cellulose synthase-like protein) were involved in the starch and sucrose metabolism pathways, and *BraA04g029950.3C* (PEPC) was involved in carbon metabolism, pyruvate metabolism, and carbon fixation in photosynthetic organisms. Previous studies have demonstrated that the plant hormone signal transduction pathway plays an important role in pollen development. The anther development of plants is not only related to the content of several endogenous hormones but also to the direct balance of various hormones^31^. *BraA07g016770.3C* (AP2), *BraA09g056770.3C* (ethylene-responsive transcription factor), *BraA07g039150.3C* and *BraA02g019090.3C* (LRR receptor-like serine/threonine-protein kinase), *BraA02g010260.3C*, *BraA09g009420.3C*, *BraA01g028580.3C*, *BraA09g061620.3C*, *BraA08g013210.3C*, and *BraA03g040360.3C* (serine/threonine-protein kinase), and *BraA06g043820.3C* (SPL) were involved in plant hormone signal transduction pathways. These results can help to further explore the molecular mechanisms of anther development in Chinese cabbage.

**Figure 7:**
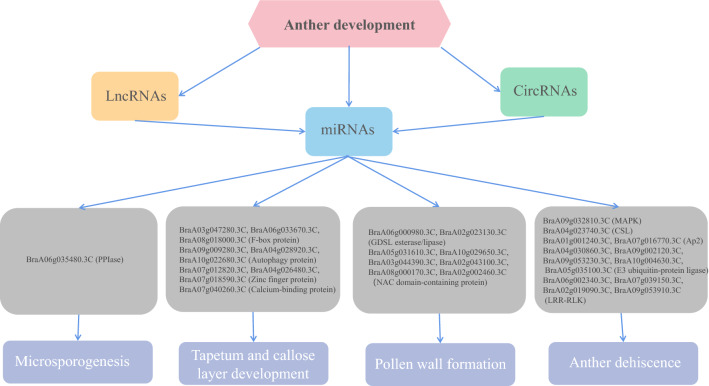
Proposed model of anther development in Chinese cabbage. Differentially expressed miRNAs (DEmiRNAs) act as key regulators that directly target important anther development-related genes. Several differentially expressed lncRNAs and circRNAs can act as ceRNAs to competitively bind to the common miRNA response elements of DEmiRNAs, which may indirectly affect the expression of differentially expressed mRNAs.

The key regulatory genes in the process of anther development have been widely reported in the model plant *Arabidopsis*, including those involved in microsporogenesis, tapetum and callose layer development, pollen wall formation, and anther dehiscence^48^. In this study, we identified a series of genes involved in anther development and their interactions with miRNAs, lncRNAs, and circRNAs (Fig. 7). Studies have demonstrated that the expression of peptidyl-prolyl cis–trans isomerase (PPIase) can affect the pollen fertility of rice by affecting microspore development^49^. In this study, the upregulated DEmRNA, *BraA06g035480.3C* (PPIase), was regulated by *bra-MIR9569-p3*, *MSTRG.11029*, *MSTRG.25631.1*, *MSTRG.5336.1*, *MSTRG.93*, *MSTRG.1825.1*, *MSTRG.19952.1*, *MSTRG.10159.1*, and *circRNA105*, which may be important for microsporogenesis in Chinese cabbage.

The tapetum is composed of a secretory cell, which is an important part of pollen formation. In the early stage of anther development, the tapetum surrounds the anther, providing various nutrients for microspore development. After meiosis of microspore cells, the tapetum secretes callose degrading enzymes, which decompose the callose wall of the cyst tetraspore, thus releasing microspores^50^. The F-box protein has been reported to play an important role in regulating tapetum degeneration and pollen maturation during *Arabidopsis* anther development^51^. Autophagy is an important cellular process that can break down cellular components during aging, starvation, and stress. In *Arabidopsis*, autophagy plays an important role in tapetum programmed cell death and pollen development^52^. Zinc finger proteins play an important role in the late development of the tapetum. Silencing of the tapetum-specific zinc finger gene (TAZ1) leads to premature degeneration and pollen abortion in Petunia^53^. Ca^2^^+^-binding proteins play an important role in regulating cell degradation and pollen formation in the rice tapetum. Ca^2+^-binding protein mutations cause delayed degeneration of tapetum cells, reduced degeneration of the callose wall around microspores, pollen abortion, and complete male sterility^54^. In the present study, *BraA03g047280.3C*, *BraA06g033670.3C*, and *BraA08g018000.3C* (F-box proteins), *BraA09g009280.3C*, *BraA04g028920.3C*, and *BraA10g022680.3C* (autophagy proteins), *BraA07g012820.3C*, *BraA04g026480.3C*, and *BraA07g018590.3C* (zinc finger proteins), *BraA07g040260.3C* (calcium-binding proteins), nine interacting miRNAs, 57 lncRNAs, and seven circRNAs are possibly involved in the development of the Chinese cabbage tapetum.

Successful male reproduction in flowering plants requires complex interactions between the anther wall layer and reproductive cells. The key to pollen development is the timely degradation of the anther wall layer and tapetum. Three GDSL esterase/lipase proteins, OsGELP34, OsGELP110, and OsGELP115, which play important roles in the formation of exine, have been identified in rice^55^. NAC domain transcription factors are plant-specific transcription regulators involved in the biosynthesis of plant secondary cell walls^56^. Here, *BraA06g000980.3C* and *BraA02g023130.3C* (GDSL esterase/lipase) are the target genes of *ath-miR845b-p3* and interact with lncRNAs (*MSTRG.9089.1*, *MSTRG.4677.1*, *MSTRG.4306.1*, *MSTRG.25939.1*, *MSTRG.21171*, *MSTRG.19614.1*, *MSTRG.19265.1*, *MSTRG.18069.1*, *MSTRG.17974.1*, *MSTRG.14213.1*, *MSTRG.1403.1*, *MSTRG.13821.1*, *MSTRG.12550.1*, *MSTRG.11661*, *MSTRG.11253.1*, *MSTRG.10845.1*, and *MSTRG.10406.1*) and circRNAs (*circRNA161*, *circRNA146*, and *circRNA106*). *BraA05g031610.3C*, encoding NAC domain-containing protein, is regulated by 21 DElncRNAs and *ath-miR829-p5*. Five other DEmRNAs encoding NAC domain-containing proteins, namely *BraA10g029650.3C*, *BraA03g044390.3C*, *BraA02g043100.3C*, *BraA08g000170.3C*, and *BraA02g002460.3C*, were the target genes of *ath-miR164a*, *bra-miR164a*, and *mtr-miR164a*, and further interacted with *MSTRG.18058.1*. These regulatory networks may participate in pollen wall formation in Chinese cabbage.

Anther dehiscence determines the success of sexual reproduction in flowering plants by releasing pollen grains during pollination and fertilization. Many AP2 genes were downregulated in the anther deficient mutants, which may be related to the regulation of anther development. The putative AP2 interacting protein is involved in many functions of development and stress response, including flower development, pathogenesis, photomorphogenesis, and plant hormone signal transduction^38^. Leucine-rich repetitive receptor-like kinase (LRR-RLK) and mitogen-activated protein kinase (MAPK) pathways have been demonstrated to regulate various aspects of plant growth and development. In *Arabidopsis*, the normal development of anthers depends on the interaction between cells so as to regulate cell proliferation and differentiation, and LRR-RLK and MAPK are involved in anther dehiscence and differentiation^57^. In rice, cellulose synthase-like (CSL) genes play a pivotal role in pollen formation, anther dehiscence, cell proliferation, stomatal formation, and cell arrangement in different tissues^58^. E3 ubiquitin-protein ligase affects the fertility of *Arabidopsis* by affecting anther dehiscence and pollen development^59^. In the present study, *BraA01g001240.3C* and *BraA07g016770.3C* (Ap2), *BraA06g002340.3C*, *BraA07g039150.3C*, *BraA02g019090.3C*, and *BraA09g053910.3C* (LRR-RLK), *BraA09g032810.3C* (MAPK), *BraA04g023740.3C* (CSL), *BraA04g030860.3C*, *BraA09g002120.3C*, *BraA09g053230.3C*, *BraA10g004630.3C*, and *BraA05g035100.3C* (E3 ubiquitin-protein ligase), and their related ceRNAs are possibly involved in anther dehiscence and differentiation.

These results extend the regulatory network of anther development by adding new regulatory ceRNA–miRNA–target gene relationships. Here, all identified lncRNAs, circRNAs, miRNAs, target genes, and their interactions provide a meaningful database for future research. However, to understand the biological process of anther development, further experimental research and computational analyses are required.

## Materials and methods

### Plant materials

In this study, the Chinese cabbage double haploid (DH) line ‘FT’, obtained from an isolated microspore culture, was used as the material^60^. The samples consisted of the following two parts. (1) On August 10, the DH ‘FT’ seeds germinated at room temperature were sown in a greenhouse of Shenyang Agricultural University after vernalization at 4 °C for 20 days. During flowering, the flower buds of different sizes were collected, the anthers were stripped out, and the anthers at different development stages were mixed and named “Ant.” (2) On August 10, the DH ‘FT’ seeds germinated at room temperature were sown in the greenhouse of Shenyang Agricultural University. When the seedlings grew to the four true-leaf stage, the entire plant was taken out and cleaned, and then the whole vegetative mass (including the root, stem, and leaf) was sampled and named “Mix.” The samples were quickly frozen in liquid nitrogen and completely ground for RNA extraction.

### RNA library construction and sequencing

Total RNA from ‘Ant’ and ‘Mix’ samples was extracted using TRIzol reagent (Invitrogen, CA, USA). RNA content and purity were analyzed using a Bioanalyzer 2100 (Agilent, CA, USA) and NanoDrop 2000 (Thermo, DE, USA). According to the TruSeq small RNA sample preparation kit (Illumina, San Diego, USA), the small RNA libraries of ‘Ant’ and ‘Mix’ were prepared with approximately 1 µg of total RNA. Then, the single terminal was sequenced on an Illumina Hiseq 2500 (LC-BIO, China).

For mRNA, lncRNA, and circRNA, the total RNA representing a specific fat type was used to consume ribosomal RNA according to the Epicenter Ribosomal RNA Zero Gold kit (Illumina). Purified poly (A)- or poly (A) + RNA fragments were cut into small pieces with divalent cations. Then, according to the mRNA-Seq sample preparation kit (Illumina), the RNA fragments were reverse transcribed to create the final cDNA library, with an average insertion size of 300 bp (± 50 bp) for the paired-end library. The paired ends were sequenced using an Illumina Hiseq 4000 (LC-BIO).

### miRNA identification and target gene prediction

After removing bases with adapter dimer and low complexity, common RNA families (tRNA, rRNA, sonRNA, and snRNA) and duplication from the raw reads, which were unique sequences of 18–25 nucleotides were mapped to specific species precursors in miRBase 21.0 to identify known and novel miRNAs. We analyzed the expression of miRNAs in ‘Ant’ and ‘Mix’ libraries and compared the two libraries in pairs to identify differentially expressed miRNAs (DEmiRNAs). When |log2(Fold change)|> 1 and *P *value ≤ 0.05, miRNA was considered to be differentially expressed between ‘Ant’ and ‘Mix’ libraries. To predict the miRNA-targeted genes and determine the miRNA binding sites, we used a target finder algorithm (Target Finder 50), and the alignment score value was used as the screening standard^61^. The miRNA and mRNA were compared, and complementary pairing in the comparitive position was scored^62^. The default maximum alignment score in this study was 4 points. GO terms and KEGG pathways of miRNA targets were also annotated. GO terms were enriched using Blast2GO by referring to the GO database^63^. KEGG pathway analysis was performed with reference to the KEGG pathway database (www.kegg.jp/kegg/kegg1.html)64.

### Identification and differential expression analysis of mRNA, lncRNA, and circRNA

The reads containing adapter contamination, undetermined bases, and low-quality bases were removed using Cutadapt^65^. FastQC was used to verify sequence quality (http://www.bioinformatics.babraham.ac.uk/projects/fastqc/). The clean reads were mapped on to the *Brassica rapa* v3.0 reference genome according to Bowtie2^66^ and Tophat2^67^, and the mapped reads were assembled using StringTie^68^. After the final transcriptome generation, the expression levels of all transcripts were estimated using StringTie and Ballgown^69^. Differentially expressed mRNAs (DEmRNAs) were identified when |log2 (fold change)|> 2 and *P* value ≤ 0.01.

Transcripts that overlapped with known mRNAs and those less than 200 bp were discarded. CNCI^70^, CPC^71^, and Pfam^72^ were used to predict transcripts with encoding potential. Transcripts with a CNCI score < 0 and a CPC score < − 1 were deleted. The rest of the class codes (I, j, o, u, x) were considered lncRNAs, which may play cis roles in adjacent target genes. To investigate the function of lncRNAs, the cis-target genes of lncRNAs were predicted. In this study, Perl script was used to screen 100 Kb upstream and downstream of coding genes. Then, we determined the functional analysis of lncRNA target genes using internal scripts. A standard of | log2 (fold change) |> 1 and *P* value ≤ 0.05 were used to screen differentially expressed lncRNAs (DElncRNAs).

A circexplorer^69,71^ was used to assemble the mapped reads to circRNAs; following this, tophat-fusion and CIRCExplorer recognized the reverse splicing reads in the unmapped reads. Based on the structural characteristics and splicing sequence characteristics of circRNAs, we used the following criteria to identify circRNAs: (1) mismatch ≤ 2; (2) back-spliced junction reads ≥ 1; and (3) distance between two splice sites < 100 Kb. The expression of circRNAs was calculated using in-house scripts. The R package edgeR^70^ was used to identify differentially expressed circRNAs (DEcircRNAs) between ‘Ant’ and ‘Mix’ libraries with the criteria of P-value ≤ 0.05 and | log2 (fold change) |> 1.

The GO terms and KEGG pathways of mRNAs, lncRNAs, and circRNAs were also annotated^63,64^.

### Construction of ceRNA network

In this study, ceRNA analysis was divided into two parts: miRNA–mRNA and miRNA–lncRNA/circRNA. Target finder software was used for miRNA–mRNA analysis, and the alignment score value was used as the screening standard. The prediction results were scored by comparing the strands of miRNA and mRNA and complementary pairing in comparative positions was scored—one point for a mismatch or missing nt, 0.5 points for a G:U pairing, and double penalty point for a G:U pairing in the core area, starting from the 2^nd^ to the 13^th^ nt of miRNA in the core area. The alignment score indicated the degree of matching between the target gene and miRNA. The lower the score, the more complete and credible the match. The default maximum penalty score in this study was four points. Search 36 (36.3.6) was used for miRNA–lncRNA/circRNA analysis with the following rules^73^: (1) bulges must be on the ncRNA and in the middle of the miRNA; (2) at most, four mismatches were allowed in positions other than the middle of the miRNA, and continuous mismatches could not exceed two; (3) bulges were not allowed in positions other than the middle of the miRNA. The software predicted the target binding relationship between miRNA and mRNA (3' UTR), lncRNA, and circRNA, and then constructed the ceRNA relationship pairs with circRNA/lncRNA–miRNA–mRNA based on common miRNA binding. The network diagram was constructed using Cytoscape (https://cytoscape.org) to visualize the regulatory relationship.

### Quantitative real-time PCR (qRT-PCR)

The expression levels of selected DEmRNAs, DEmiRNAs, DEcircRNAs, and DElncRNAs were validated by qRT-PCR. Total RNAs and small RNAs of ‘Ant’ and ‘Mix’ were extracted using an RNApure Total RNA Kit (Aidlab, RN03, China). RNA was reverse transcribed using an Evo M-MLV RT Kit II (Accurate Biotechnology, AG11711, China). Oligo dT primers and random 6-mer primers were used to construct the 1st strand cDNA for quantitative analysis of mRNAs and lncRNAs. The miRNAs were reverse-transcribed using the downstream primers of U6 endogenous reference gene and the specific stem-loop primers. The circRNAs were reverse-transcribed using the qRT‒PCR downstream primers and random 6-mer primers. qRT-PCR was performed using a SYBR® Green Premix Pro Taq HS qPCR Kit (Accurate Biotechnology) with an ABI 7300 instrument (Thermo Fisher Scientific, Waltham, MA, USA). The qRT-PCR procedure was as follows: 50 °C for 2 min; 95 °C for 30 s; 40 cycles at 95 °C for 5 s and 60 °C for 30 s; 95 °C for 15 s; 60 °C for 1 min; and 95 °C for 15 s. U6 was used as an endogenous control for the miRNAs. Actin was used as an endogenous control for circRNAs, lncRNAs, and mRNAs. All qRT-PCR reactions were performed in three technical replicates and three biological replicates. The primer sequences are listed in Table S12. RT is the specific stem-loop primers used to amplify miRNAs.

### Promoter activity assay

The promoter region, which was 2000 bp upstream of the anther predominant expression gene, was amplified and a recombinant plasmid was obtained by connecting the target fragment with a pC1301IgT vector and transferred into *Escherichia coli* DH5 α competent cells. The expression vector was transformed into *Agrobacterium tumefaciens* GV3103 competent cells and was introduced into wild-type *Arabidopsis* according to the *Agrobacterium*-mediated floral-dip method^74^. The positive transgenic *Arabidopsis* was separately screened on MS solid medium containing 30 μg/mL hygromycin and then validated using PCR. Finally, GUS staining was performed on transgenic plants^75^.

### Ethics approval and consent to participate

The current study complies with relevant institutional, national, and international guidelines and legislation for experimental research and field studies on plants (either cultivated or wild), including the collection of plant material.

## Supplementary Information

Supplementary Information 1.

Supplementary Information 2.

Supplementary Information 3.

Supplementary Information 4.

Supplementary Information 5.

Supplementary Information 6.

Supplementary Information 7.

Supplementary Information 8.

Supplementary Information 9.

Supplementary Information 10.

Supplementary Information 11.

Supplementary Information 12.

Supplementary Information 13.

Supplementary Information 14.

Supplementary Information 15.

Supplementary Information 16.

Supplementary Information 17.

## Data Availability

The datasets supporting the conclusions of this article are included within the article and its additional files. The transcriptome sequencing data were deposited in the National Center for Biotechnology Information (NCBI) Gene Expression Omnibus (GEO) Database under accession number GSE171705 and GSE171706. Genomic sequences and gene annotation information of *Brassica rapa* were downloaded online at http://brassicadb.cn/#/.
