# Supplementary figures and images for: Whole-transcriptome analysis and construction of an anther development-related ceRNA network in Chinese cabbage (Brassica campestris L. ssp. pekinensis)
Source: Sci Rep. 2022 Feb 17;12:2667. doi: 10.1038/s41598-022-06556-2 (PMC8854722; doi:10.1038/s41598-022-06556-2)

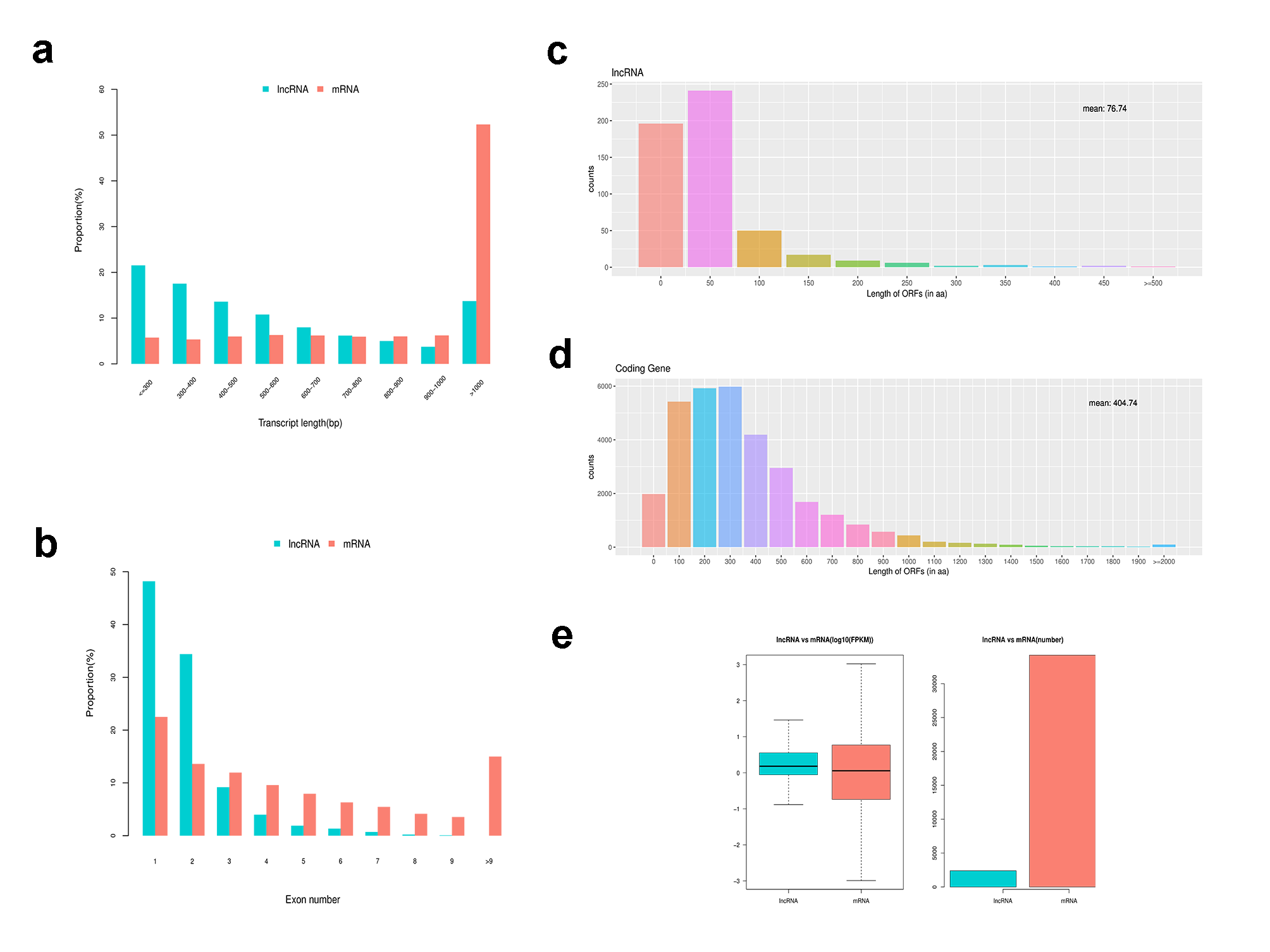

Supplement: Supplementary file 1 — Supplementary Information 1. [file 41598_2022_6556_MOESM1_ESM.tif]

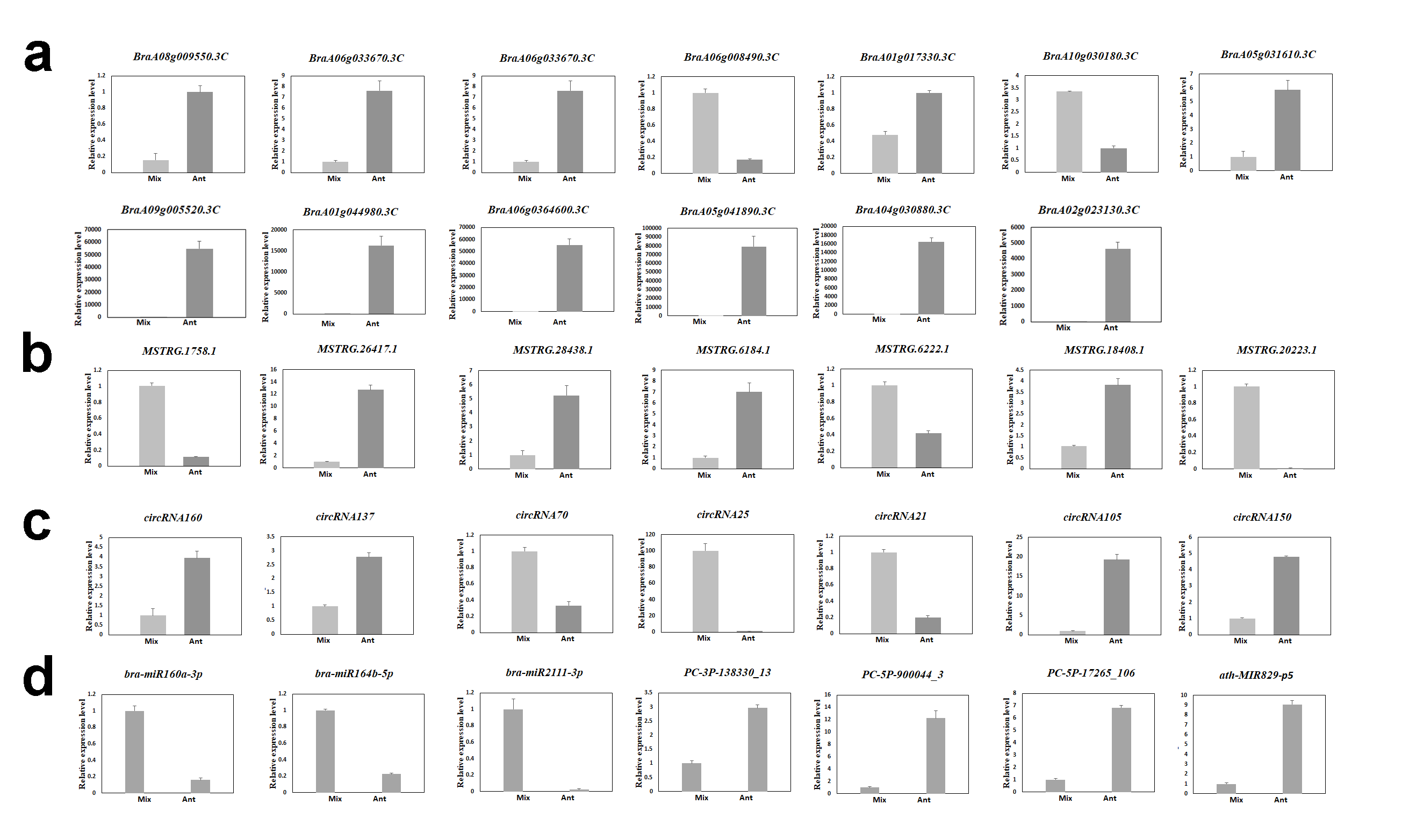

Supplement: Supplementary file 2 — Supplementary Information 2. [file 41598_2022_6556_MOESM2_ESM.tif]

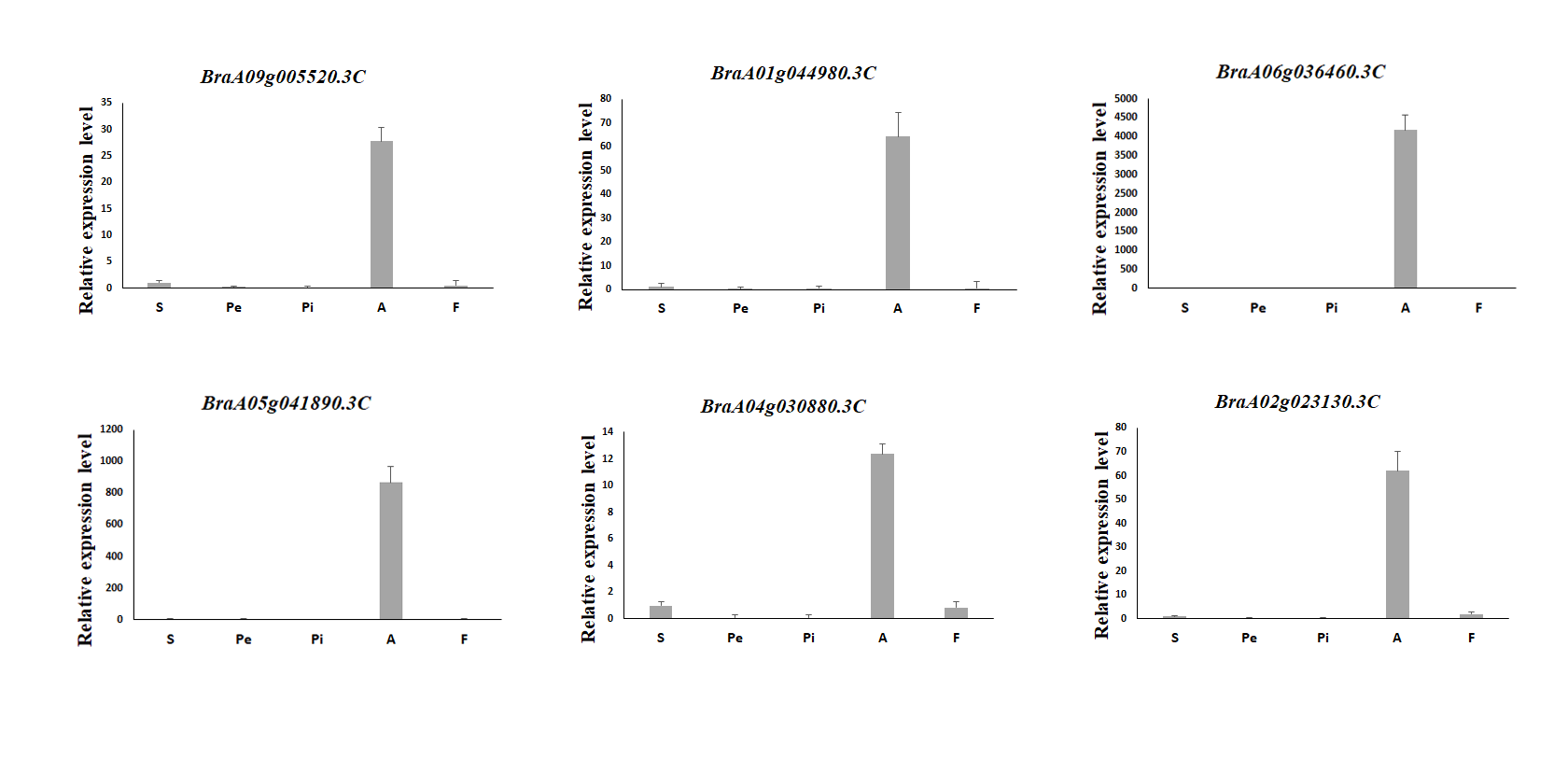

Supplement: Supplementary file 3 — Supplementary Information 3. [file 41598_2022_6556_MOESM3_ESM.tif]
