# Supplementary material for: Whole-transcriptome analysis and construction of an anther development-related ceRNA network in Chinese cabbage (Brassica campestris L. ssp. pekinensis)
Source: Sci Rep. 2022 Feb 17;12:2667. doi: 10.1038/s41598-022-06556-2 (PMC8854722; doi:10.1038/s41598-022-06556-2)
Supplement: Supplementary file 4 — Supplementary Information 4. [file 41598_2022_6556_MOESM4_ESM.docx]

**Supplementary figures and tables legends**

**Fig. S1** Comparison of structural characteristics and expression levels of lncRNA with mRNA. (a) transcript length distribution of lncRNA and mRNA; (b) exon number of lncRNA and mRNA; (c), (d) ORF length distribution of lncRNA and mRNA; (e) expression levels of lncRNA and mRNA.

**Fig. S2** qRT-PCR analysis of differentially expressed mRNAs (a), lncRNAs (b), circRNAs (c), and miRNAs (d) in anther (‘Ant’), and vegetative mass of four true leaves (‘Mix’) samples.

**Fig. S3** qRT-PCR analysis of six differentially expressed mRNAs in different floral organs. S: sepal, Pe: petal, Pi: pistil, A: anther, F: filament.

**Table S1**: Summary of valid data from the anther (‘Ant’), and vegetative mass of four true leaves (‘Mix’) sRNA libraries.

**Table S2**: Length and count characteristics of the sequencing results.

**Table S3**: Summary of total miRNA, mRNA, lncRNA, and circRNA identified in this study.

**Table S4**: Summary of miRNA families identified in this study.

**Table S5**: Summary of differentially expressed (DE) miRNAs, mRNAs, lncRNAs, and circRNAs identified in this study.

**Table S6**: Summary of miRNA target gene prediction.

**Table S7**: List of GO terms for differentially expressed (DE) miRNAs, mRNAs, lncRNAs, and circRNAs.

**Table S8**: Kyoto Encyclopedia of Genes and Genome (KEGG) pathway assignments for differentially expressed (DE) miRNAs, mRNAs, lncRNAs, and circRNAs.

**Table S9**: Summary of the RNA sequencing data.

**Table S10**: Co-expression relationship of lncRNAs and mRNAs.

**Table S11**: Summary of ceRNA relationships.

**Table S12**: Primers used for qRT-PCR.
