# Supplementary material for: Whole-transcriptome analysis and construction of an anther development-related ceRNA network in Chinese cabbage (Brassica campestris L. ssp. pekinensis)
Source: Sci Rep. 2022 Feb 17;12:2667. doi: 10.1038/s41598-022-06556-2 (PMC8854722; doi:10.1038/s41598-022-06556-2)
Supplement: Supplementary file 5 — Supplementary Information 5. [file 41598_2022_6556_MOESM5_ESM.docx]

**Supplementary figures**

**
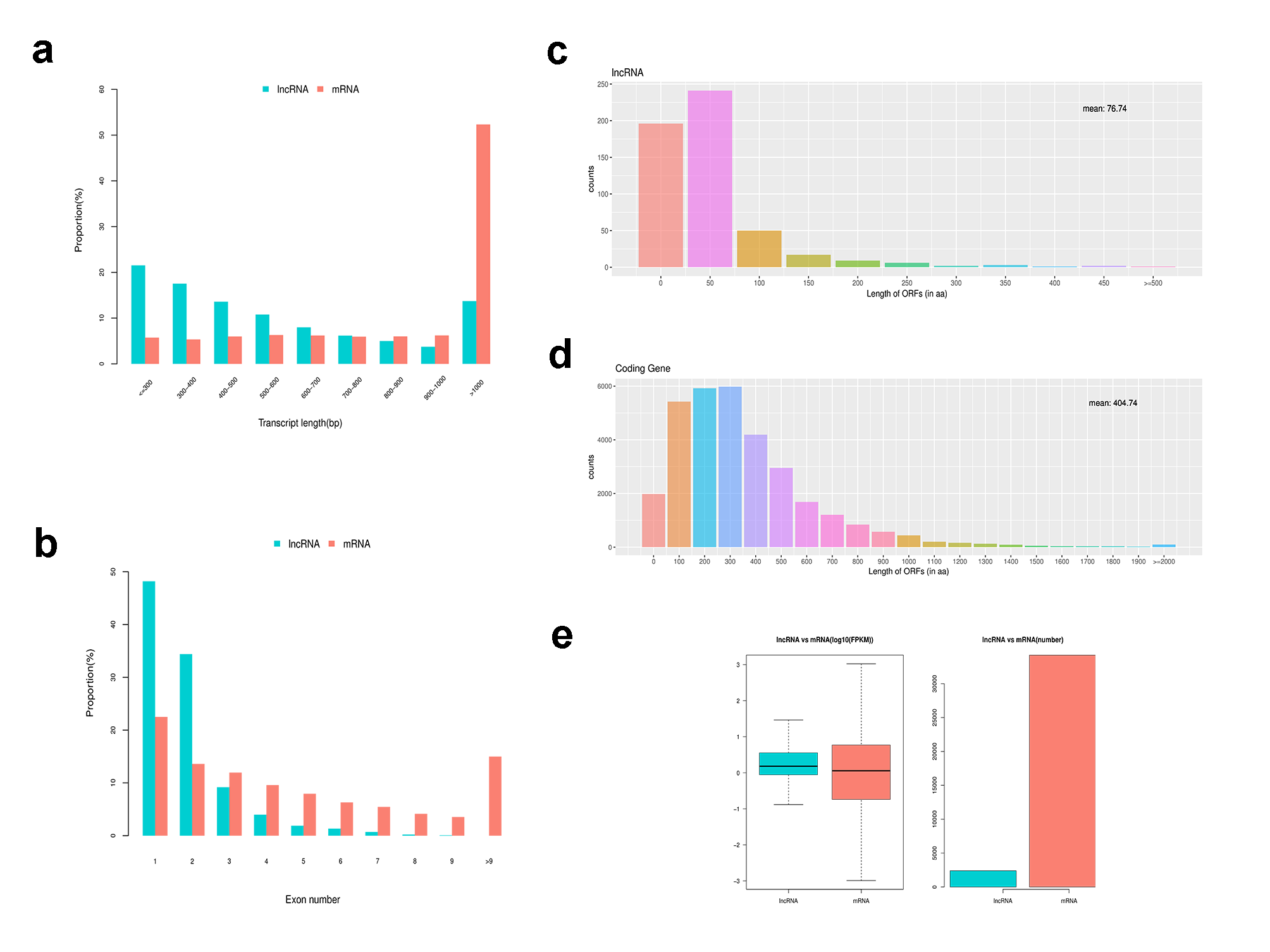
**

**Fig. S1** Comparison of structural characteristics and expression levels of lncRNA with mRNA. (a) transcript length distribution of lncRNA and mRNA; (b) exon number of lncRNA and mRNA; (c), (d) ORF length distribution of lncRNA and mRNA; (e) expression levels of lncRNA and mRNA.


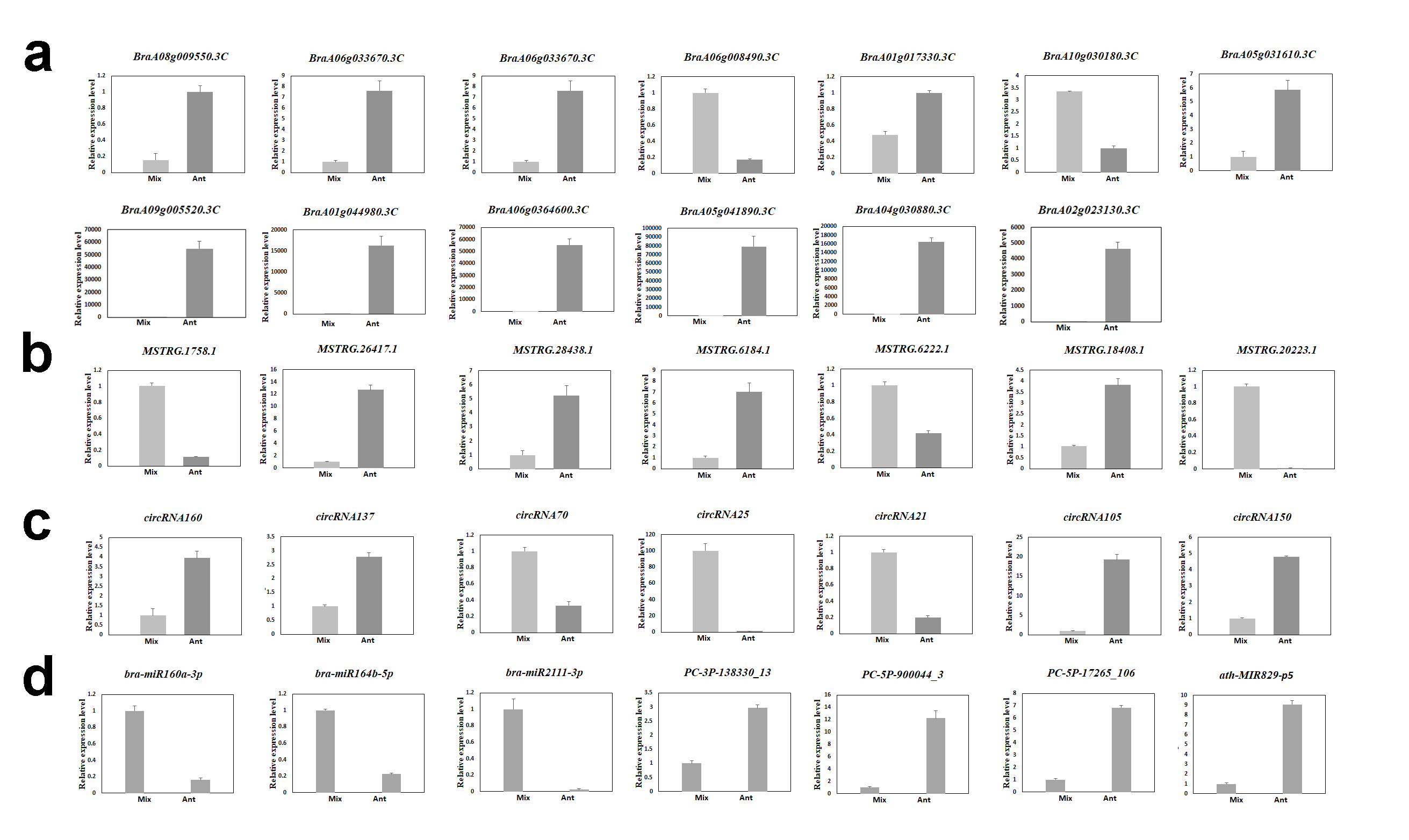


**Fig. S2** qRT-PCR analysis of differentially expressed mRNAs (a), lncRNAs (b), circRNAs (c), and miRNAs (d) in anther (‘Ant’), and vegetative mass of four true leaves (‘Mix’) samples.


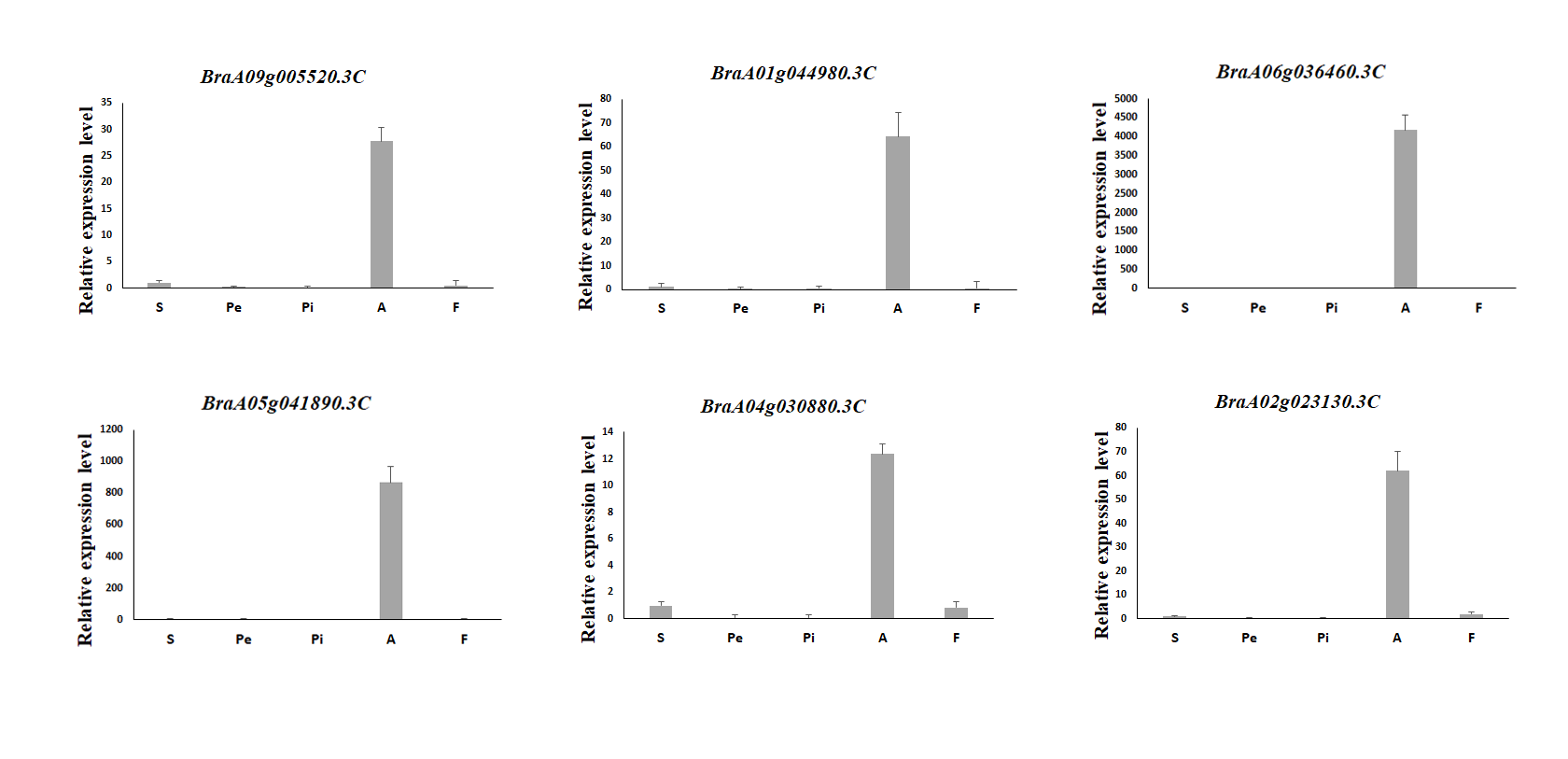


**Fig. S3** qRT-PCR analysis of six differentially expressed mRNAs in different floral organs. S: sepal, Pe: petal, Pi: pistil, A: anther, F: filament.
