# Supplementary material for: Whole-transcriptome analysis and construction of an anther development-related ceRNA network in Chinese cabbage (Brassica campestris L. ssp. pekinensis)
Source: Sci Rep. 2022 Feb 17;12:2667. doi: 10.1038/s41598-022-06556-2 (PMC8854722; doi:10.1038/s41598-022-06556-2)
Supplement: Supplementary file 6 — Supplementary Information 6. [file 41598_2022_6556_MOESM6_ESM.docx]

**Table S1** Summary of valid data from the anther (‘Ant’), and vegetative mass of four true leaves (‘Mix’) sRNA libraries

|  | Mix | | | | Ant | | | |
| --- | --- | --- | --- | --- | --- | --- | --- | --- |
|  | Total | % of Total | Unique | % of Unique | Total | % of Total | Unique | % of Unique |
| Raw reads | 22291255 | 100.00 | 6231290 | 100.00 | 24706757 | 100.00 | 8221376 | 100.00 |
| 3ADT&length filter | 7481322 | 33.56 | 1501258 | 24.09 | 8554172 | 34.62 | 2180799 | 26.53 |
| Junk reads | 76680 | 0.34 | 56039 | 0.90 | 150809 | 0.61 | 99477 | 1.21 |
| Rfam | 1438500 | 6.45 | 115116 | 1.85 | 1133393 | 4.59 | 71058 | 0.86 |
| mRNA | 1037508 | 4.65 | 335078 | 5.38 | 1554315 | 6.29 | 444538 | 5.41 |
| Repeats | 57038 | 0.26 | 9939 | 0.16 | 44401 | 0.18 | 11790 | 0.14 |
| Valid reads | 12257469 | 54.99 | 4222985 | 67.77 | 13351418 | 54.04 | 5419915 | 65.92 |
