# Supplementary material for: Whole-transcriptome analysis and construction of an anther development-related ceRNA network in Chinese cabbage (Brassica campestris L. ssp. pekinensis)
Source: Sci Rep. 2022 Feb 17;12:2667. doi: 10.1038/s41598-022-06556-2 (PMC8854722; doi:10.1038/s41598-022-06556-2)
Supplement: Supplementary file 7 — Supplementary Information 7. [file 41598_2022_6556_MOESM7_ESM.docx]

|  | Mix | | | | Ant | | | |
| --- | --- | --- | --- | --- | --- | --- | --- | --- |
| Length | Total | % of Total | Unique | % of Unique | Total | % of Total | Unique | % of Unique |
| 18 | 639722 | 5.22 | 127503 | 3.02 | 166943 | 1.25 | 51890 | 0.96 |
| 19 | 767257 | 6.26 | 174116 | 4.12 | 284211 | 2.13 | 107227 | 1.98 |
| 20 | 1037156 | 8.46 | 227599 | 5.39 | 685082 | 5.13 | 198606 | 3.66 |
| 21 | 2771419 | 22.61 | 435640 | 10.32 | 3429790 | 25.69 | 650010 | 11.99 |
| 22 | 1359015 | 11.09 | 402555 | 9.53 | 1847355 | 13.84 | 700536 | 12.93 |
| 23 | 1617763 | 13.20 | 788618 | 18.67 | 1935872 | 14.50 | 907564 | 16.74 |
| 24 | 3604278 | 29.40 | 1920445 | 45.48 | 4753412 | 35.60 | 2705596 | 49.92 |
| 25 | 460859 | 3.76 | 146509 | 3.47 | 248753 | 1.86 | 98486 | 1.82 |
| Valid reads | 12257469 |  | 4222985 |  | 13351418 |  | 5419915 |  |

**Table S2** The length and counts characteristics of the sequencing results
