# Supplementary material for: Whole-transcriptome analysis and construction of an anther development-related ceRNA network in Chinese cabbage (Brassica campestris L. ssp. pekinensis)
Source: Sci Rep. 2022 Feb 17;12:2667. doi: 10.1038/s41598-022-06556-2 (PMC8854722; doi:10.1038/s41598-022-06556-2)
Supplement: Supplementary file 14 — Supplementary Information 14. [file 41598_2022_6556_MOESM14_ESM.docx]

**Table S9** Summary of the RNA-seq data

| Sample name | Mix | Ant |
| --- | --- | --- |
| Raw reads | 141748652 | 139573514 |
| Raw bases | 21.26G | 20.94G |
| Valid reads | 139969178 | 136757604 |
| Valid bases | 21.00G | 20.51G |
| Q20(%) | 99.66 | 99.64 |
| Q30(%) | 95.97 | 96.26 |
| GC(%) | 44 | 44 |
| Total mapped reads | 128848721 (92.06%) | 121717101 (89.00%) |
| Multiple mapped reads | 36084886 (25.78%) | 30184399 (22.07%) |
| Unique mapped reads | 92763835 (66.27%) | 91532702 (66.93%) |
| Non-splice reads | 96256969 (68.77%) | 74279988 (54.32%) |
| Splice reads | 20130727 (14.38%) | 40654446 (29.73%) |
| Valid Ratio | 98.74% | 97.98% |
| PE mapped reads | 120946664 (86.41%) | 113661104 (83.11%) |
| Reads map to sense strand | 57824573 (41.31%) | 57291787 (41.89%) |
| Reads map to antisense strand | 58563123 (41.84%) | 57642647 (42.15%) |
